# Templated freezing: a simple method may increase gripping force of the clamp on the tendon

**DOI:** 10.1186/s13018-022-03209-y

**Published:** 2022-06-15

**Authors:** T. Wang, H. Yu

**Affiliations:** 1 Graduate School, Shenyang Medical College, No.5 South Qi West Road, North Huanghe Street, Shenyang, 110000 China; 2 Department of Orthopedics, The First Hospital of China Medical University, 155 Nanjing North Street, Shenyang, 110000 China

**Keywords:** Biomechanical phenomena, Mechanical tests, Tensile strength, clamping, Tendon

## Abstract

**Purpose:**

To evaluate the effectiveness of combining a customized mold with frozen conventional clamps against other freezing and non-freezing methods.

**Methods:**

Forty-five porcine and 45 chicken tendons were evenly divided into five groups (*n* = 9 + 9/group): control group, non-freezing with gauze placed between tendon and clamp (gauze), non-freezing with suture fixation at tendon ends (suture), freezing with dry ice pocket placed at the clamps (pocket), and freezing using a templated liquid nitrogen clamp with a customized mold (mold). Tension tests were used to measure failure modes and loads.

**Result:**

Slippage and avulsion were observed in non-freezing groups with significantly lower failure loads compared to freezing methods. With freezing, rupture occurred near the central point only in the mold group. The failure loads for porcine tendons in the mold group were higher (2121.651 ± 73.101 N) than the pocket group (1746.337 ± 68.849 *N*). The failure loads of chicken tendons in the mold (243.552 ± 15.881 *N*) and pocket groups (260.647 ± 22.161 *N*) were not statistically different.

**Conclusion:**

Freezing clamps represent the better choice for soft tissue clamping. The customized mold method could improve gripping effectiveness.

**Supplementary Information:**

The online version contains supplementary material available at 10.1186/s13018-022-03209-y.

## Introduction

The fixation of tendons is key point during the tension test for the mechanical properties of tendons [1–3]. If slippage or damage of the tendon occurred in the clamping position during the test, it would lead to errors and affect the accuracy of conclusions about its mechanical properties. The style of tendon fixation on the clamps can be divided into freezing and non-freezing treatments. Non-freezing treatments include modifying the clamp interface geometry [4, 5], or adding additional materials [5, 6]. The modified surface of the clamps can increase the contact area and friction between tendon and clamp, but the equipment fabrication and application techniques are highly demanding. Adding additional materials such as sandpaper, adhesive and winding as the interposition only applies force to the superficial layers which may cause the inner fibers to displace and increase transverse shear forces. The slippage caused by low friction between the clamp and the soft, wet collagen tissue can easily damage the orientation of tendons due to extrusion from the clamps and reduce their effectiveness [4, 7, 8]. Freezing treatment has long been considered the gold standard for high load mechanical testing of soft tissues, [5, 9]. It has been shown that frozen tendons are stiffer and more rigid, which can prevent damage to the tendon and slippage between the tendon and clamps [10]. Freezing methods include cryogenic and thermoelectric approaches [11]. The former method applies cold resources such as liquid carbon dioxide (CO2), dry ice, or liquid nitrogen [3, 9] to freeze the clamped ends of tendons. A thermoelectric approach uses a cooler to remove heat from the contact surface to achieve freezing, However, it requires long preparation time, which could be a problem for a large number of tests [5]. Recently, Hangody [11] reported a simple, convenient and enlightening method using pockets of dry ice on both sides of the clamps as a freezing method. This method provided a satisfactory outcome, but still required a modified clamp, a non-frozen asymmetrical nylon teeth jaw and an alloyed titanium clamp body designed by Shi et al. for clamping tendons(4). Due to the high cost of cryogenic and thermoelectric approaches and the time-consuming process to customize the specialized clamps, we hope to explore a relatively simple and affordable alternative to make the conventional mechanical clamps that can achieve the clamping quality similar to cryogenic and thermoelectric approaches. Accordingly, we designed a freezing method using conventional (non-customized) clamps with a simple mold. The purpose of our study was to (1) explore if the new method could provide sufficient efficacy for mechanical tendon testing, and (2) compare its effectiveness against other freezing and non-freezing methods.

## Materials and methods

### Animals and preparation

**Fig. 1:**
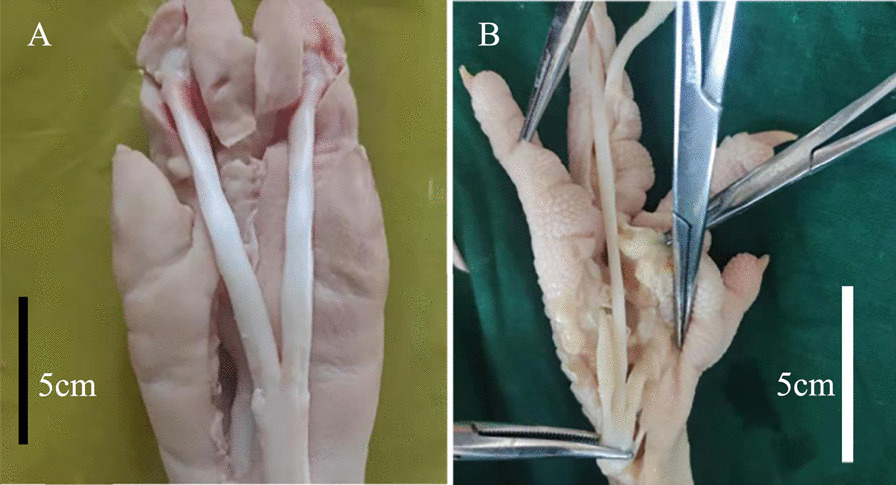
Acquisition of porcine and chicken tendons. **A** The porcine flexor digitorums tendons were removed from the attachment of phalangette to the cross of tendons. Each porcine hind-leg can get two flexor digitorum profundus tendons which were about 8–10 cm. **B** The chicken flexor digitorums tendons were removed from the cross of tendons to the attachment of the third digit. The rest of the soft tissue around the tendon needs to be removed carefully and the tendons were irrigated with saline to keep the surface moist

**Fig. 2:**
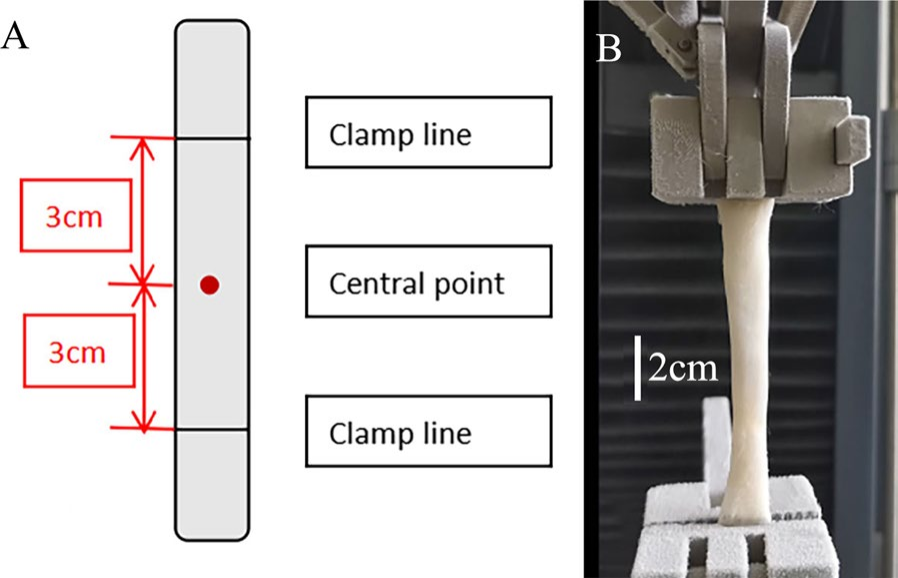
Tendon marking and installation. **A**. The central point is defined as the right middle of the tendon and the clamp lines are defined as the location where the clamp applying on the tendon. We set the distance between the center point and the clamping line at both ends to be 3 cm to keep the distance between the clamp lines at both ends to be 6 cm. **B**. The porcine tendon in pocket group. The tendon was placed parallel to the direction of moving. The clamps are fixed in the position of clamp line. After the pocket was released, the surface of the clamps appeared hoarfrost quickly, which proves that the surface temperature of the clamps has dropped below 0 ℃, and the clamps and the tendon are frozen firmly

Flexor digitorum profundus tendons from 45 porcine hind-legs and chicken feet were sourced from a local butcher (Fig. 1). The chicken tendons were acquired from the third digit, which was the longest and therefore considered more suitable for study [12, 13]. The cross section area (CSA) was calculated by approximating the cross section to an ellipse [14, 15] (*S* = Πab/4, where a and b are the average values of major and minor axes of the ellipse measured three times by a vernier-caliper with a precision of 0.02 mm, which was placed vertically to the long axis of the tendon). The CSA of tendons of porcine (23.544 ± 4.450 mm^2^) and chicken (2.076 ± 0.511mm^2^) is significantly different, representing two different sizes of tendons to simulate the different sizes encountered in the clinic [16]. After the tendons were taken, some uniform marks, including the central point and the clamp line, were made on the tendons in the same location to ensure the areas of freeze and test were the same (Fig. 2A). The central point indicated the middle of the tendon, and the clamp line, specified the location of the clamps during the test. These marks were determined with calipers (accuracy 1 mm).

**Fig. 3:**
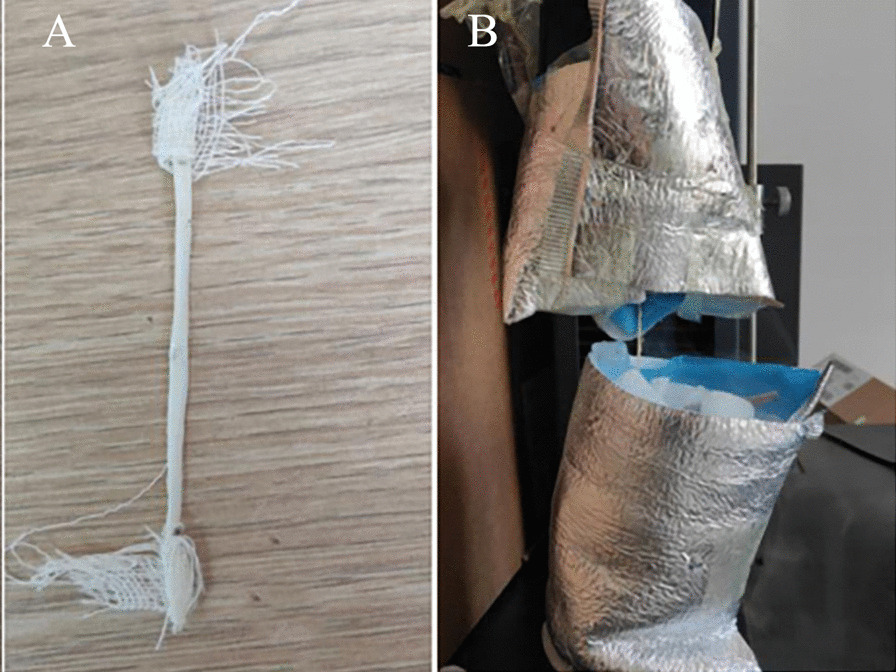
Gauze group and pocket group of chicken tendons. **A**. Gauze group, the tendons were wrapped around the outside of the clamping line for two circles and then tied firmly, trying to increase the friction between the gauze and the clamping surface of the clamp through the uneven surface of the gauze. After cutting off the excess gauze, the tendons were installed in the clamp for testing **B**. Pocket group. Pockets are made of non-woven fabric encircled by an insulation layer composed of aluminum foil, thickened polyethylene(PE) and pearl cotton are fixed to clamps on both sides. Keep plenty of dry ice in the pocket to provide a low-temperature environment

**Fig. 4:**
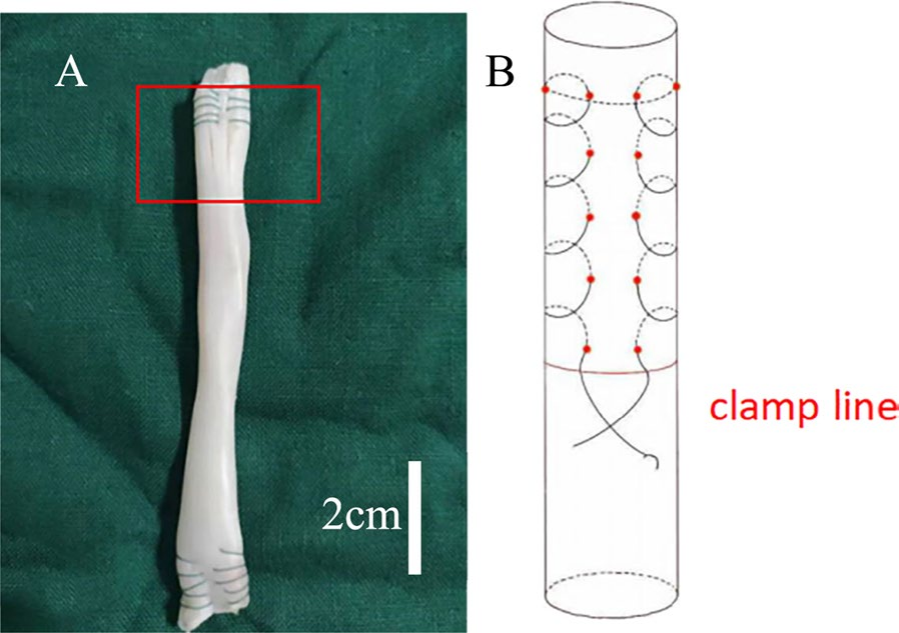
Tendons in suture group. **A**. The tendon shown above is suture group’s tendon of porcine. Suture was performed on the lateral side of the clamp line. There are five sutures perpendicular to the longitudinal axis of the tendon on the left and right sides of each end of the tendon to increase the friction. The processing method of suture group’s tendon of chicken was same as the porcine’s. **B**. The diagram of stitching method is in the red box of A. The red line is clamp line and the black curve is the suture. The solid line represents that the suture is visible on the front of the tendon, and the dotted line represents that the suture is on the back of the tendon or through the inside of the tendon. The red dot marks the needle position

**Fig. 5:**
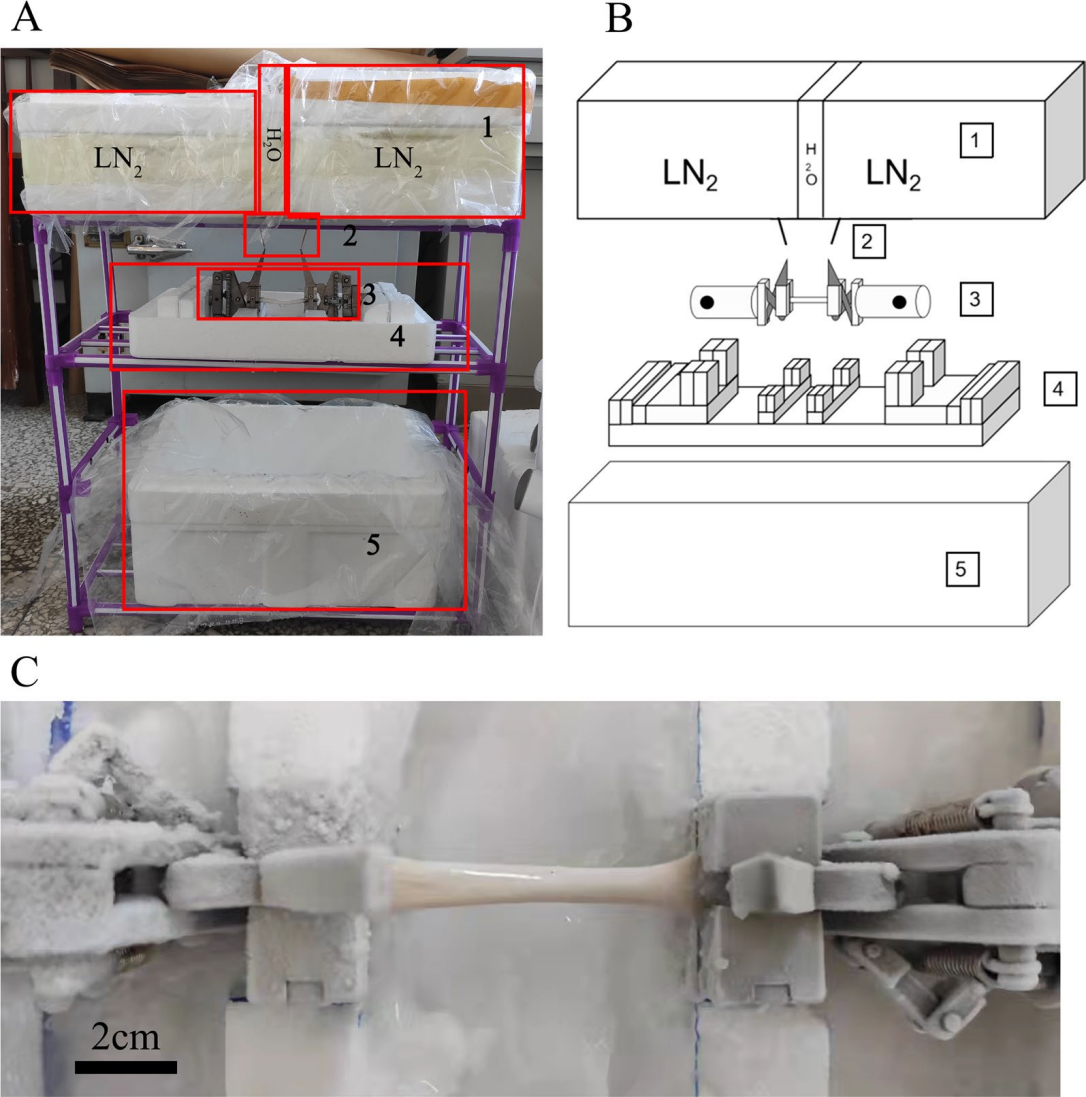
Schematic diagram of special mold **A** Overview of mold freezing system. 1.boxes containing liquid nitrogen and warm water 2.drainage rod 3.clamps with tendon 4.mold 5.collection box **B**. 1.boxes containing liquid nitrogen and warm water 2.drainage rod 3.clamps with tendon 4.mold 5.collection box. The whole system consists of three layers. The top layer is two boxes containing liquid nitrogen, sandwiched by boxes containing warm water. The middle layer is the mold with clamps, and holes shall be drilled on the mold to ensure liquid infiltration. The bottom layer is a collection box to collect the liquid flowing from the middle layer. The bottom ends of the three boxes on the top layer shall be perforated, and drainage rods shall be installed next to the bottom holes of the liquid nitrogen boxes at both ends to make the liquid nitrogen flow to the clamp along the drainage rods. The central warm water box is perforated in the middle so that it is located on the same vertical line as the center point of the tendon in middle layer. **C**. The clamping part of the tendon in the mold group was completely frozen, but the test part was not frozen

Five different treatments were used to test the tendons using the general manual metal clamp, which was made of metal with four-sided pyramid structures in the cross-sectional view. Only one pair of clamps were used in all tests. Nine porcine tendons and nine chicken tendons were included in each group. The intact tendons without any additional treatment were used as the control group. The ends of tendons were wrapped with a layer of gauze in gauze group (Fig. 3A). The gauze represents a method of adding movable high friction coefficient objects to the clamp and tendon. The tendons from the suture group were sutured with 4–0 medical thread (TianHe, China, M47193T(T-451)) at the part outside the clamp line according to the method shown in Fig. 4. There were five sutures perpendicular to the longitudinal axis of the tendon on two sides of each end of the tendon, to increase the friction. The pocket group was based on Hangody’s method [11]. Two non-woven fabric pockets with dry ice were placed on both sides of each clamp. In order to ensure the effectiveness of cold insulation, we encircled the surface of the pocket with an insulation layer composed of aluminum foil, thickened polyethylene (PE), and pearl cotton. Then, the clamp with the tendon was frozen for about 3 min according to the Hangody’s method as the study had shown that freezing for 3–5 min can achieve a firm freeze [9, 11] (Fig. 3B). In the mold group, we clamped the tendon at the clamp lines and aligned the center of the clamps with the central point of the tendon. Then, we placed the clamps and the tendon in a specified position using the specially made mold (Fig. 5). Liquid nitrogen flowed to the surface of the clamp along the drainage rod and the edge of the clamp at the clamping line, while warm water (30 ℃) flowed continuously at the center of the tendon to prevent freezing. This process was run for five minutes before stopping the liquid nitrogen. Warm water continued to flow for another minute. Then, the clamps were taken out with cotton gloves for failure testing. (Additional file 1)

### Biomechanical testing

**Fig. 6:**
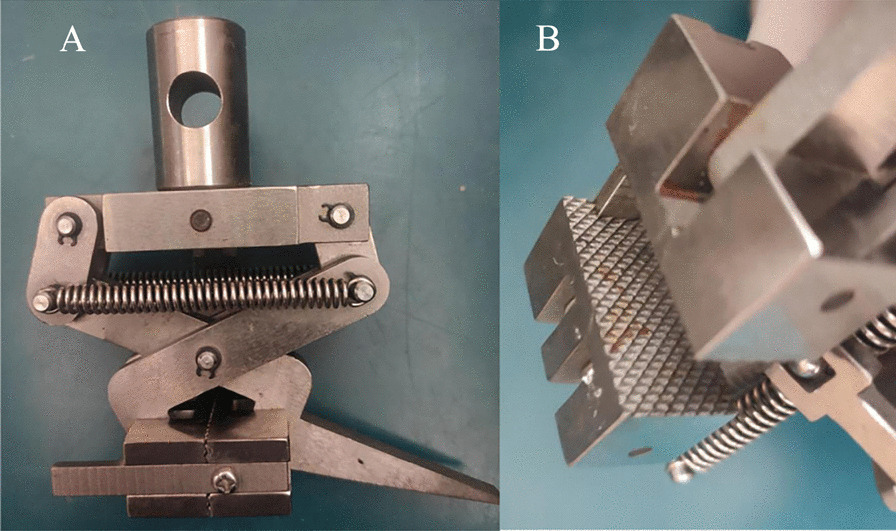
Non-special designed clamps. The clamp used is a metal clamp operated manually with four-sided pyramid structures in cross-sectional view, which is provided by Wance for the testing machine. **A** The front view of metal clamp. **B** The cross-sectional view of the clamp with four-sided pyramid structures

A microcomputer-controlled electromechanical universal testing machine (Wance, China, ETM203A) was used for failure testing. The tendons gripped on clamp lines at both ends by non-special designed clamps (Fig. 6) were tested at a speed of 20 mm/min until they reached failure point, which was defined as either significantly slippage (or avulsion) or rupture of the tendon. When a tendon failed, the morphological change of the tendon represented the failure mode and the maximum force before failure was the failure load, which could be recorded by specialized software (Wance, China, TestPilot_E10C). If the tendon failure mode was slippage, the failure load represented the fixation capacity of the clamps. When a rupture occurred, it meant that the force loaded by the machine exceeded the stress of a tendon to maintain its integrity, indicating that the fixation capacity of the fixture was higher than the failure load. During the test, the tendon was kept parallel to the moving direction of the clamps to avoid forces in other directions affecting the failure mode (Fig. 2B). Keeping the surface of the tendons moist was important during the test because an excessively dry surface could lead to desiccation, which may affect the mechanical properties of the tendons [1, 17].

### Statistical evaluation

Statistics were performed using SPSS Version 22. Differences in tendon failure loads between groups were compared using t tests.

## Results

### Failure mode

**Fig. 7:**
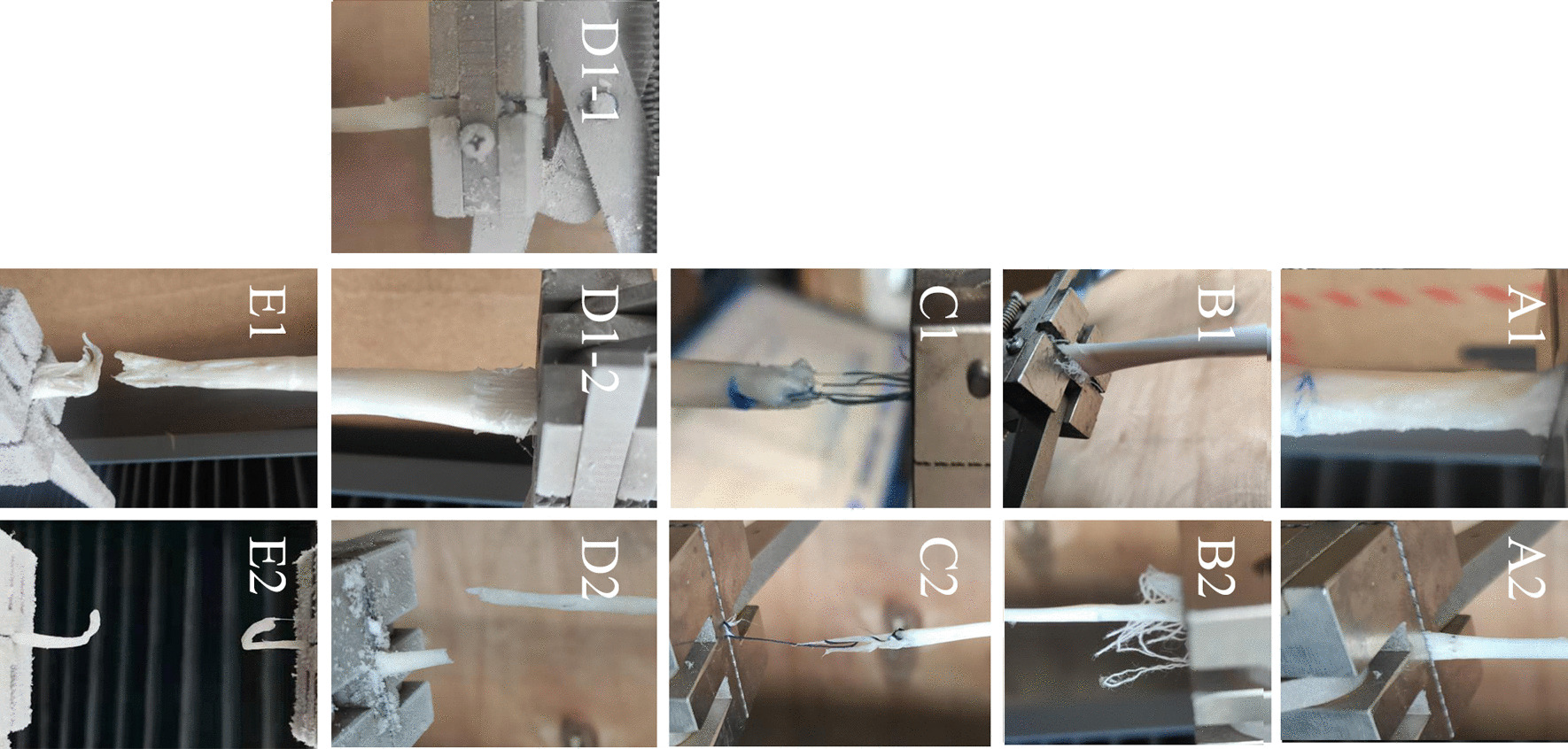
Failure mode of tendons in all groups. **A** control group **B** gauze group **C** suture group **D** pocket group **E** mold group. The number 1 represents the porcine tendon and the number 2 represents the chicken tendon. Among them, there are two failure modes of porcine tendon in Pocket group, coded as **D1-1** and **D1-2**. Control group showed slippage and avulsion (**A1** and **A2**). Gauze group was mainly manifested as slippage between tendon and gauze, and the degree of avulsion was less than control group (**B1** and **B2**). Suture group is mainly manifested as the avulsion of the suture part (**C1** and **C2**), and the tendon stump in the clamp is connected to the detached tendon by suture. Pocket group is manifested as slippage with rupture (**D1**-**1**) or slippage only (**D1**-**2**) in porcine tendon and rupture (**D2**) in chicken’s tendon. Tendon rupture (**E1** and **E2**) in mold group from both chicken’s and porcine mold group occurred in the unfrozen portion of the tendon

The observed failure modes include slippage, avulsion, and rupture. Slippage means the tendon part is out of the clamp partly, or completely. It can be seen that the clamp is obviously out of the clamp line. Avulsion is tear of tendon. It should be noted that slip allows a small amount of tissue damage, caused by clamping teeth on the tendon surface of the clamping part. If the injury causes the tear of tendon, it is avulsion. Rupture means the tendon is divided into two parts completely at the test area. (Fig. 7). In order to test the true mechanical failure of the tissue, a firm and effective grip is indicated when the only failure mode of the tendon is rupture. In the control group, slippage occurred in the porcine tendon, while avulsion occurred at the clamp line occurred in chicken tendon. (Fig. 7A). In the gauze group, the gauze was always kept between the clamps. The tendon tissue detached from the gauze, and the tearing of the tissue surface was not obvious. This indicates that while the uneven surface may indeed play a role in increased friction, this failure was caused by insufficient friction between the gauze and the tissue (Fig. 7B). In the suture group, avulsion at the clamping area occurred, and tearing of the suture was also observed in the clamping area. The suture and a small amount of tissue were detached from the tendon and adhered to the clamp face (Fig. 7C). In the pocket group, the porcine tendons showed slippage from the clamp line or rupture inside the clamp area which may also be caused by slippage, while the chicken tendons showed rupture only near the central point (Fig. 7D). There was no significant visual slippage that happened before rupture, and the rupture position was near the central point of the tested area in both porcine and chicken tendon in the mold groups (Fig. 7E).

### Failure load

**Fig. 8:**
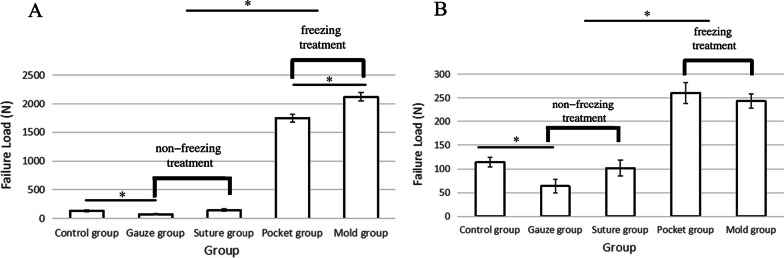
failure load of (**A**) porcine and (**B**) chicken’s tendons with different treatments. * *p* < 0.05 indicates significant differences between two groups. In all treatments, the failure load of porcine tendons was higher than chicken tendons. The failure load of freezing treatment was significantly higher than non-freezing treatment and the failure load of gauze group was significantly lower than the control group for both porcine and chicken tendons. The failure load of the mold group was significantly higher than the pocket group for porcine tendons

Failure load is the amount of force required for a tendon to reach its failure point.. The results of untreated tendons show that the test ended due to slippage with an average of only 135.654 ± 14.140 *N* in porcine tendons and 113.780 ± 10.135 *N* in chicken tendons (Table1). Overall, the gauze group for both sizes of tendons showed the lowest failure load, which was significantly lower than the control group (*p* < 0.05), with 74.310 ± 12.707 *N* in porcine and 63.903 ± 14.616 *N* chicken tendons (Table 1, Fig. 8). Sutures may change the smoothness of tissue surface and increase the friction of tissue surface. The failure load reached 144.991 ± 21.064 *N* and 101.774 ± 16.623 *N* in porcine and chicken tendons, respectively, and there was no significant difference compared with the control group (Table 1). Finally, the average failure loadforce for the porcine tendons was 1746.337 ± 68.849 *N* in the pocket group and 2121.651 ± 73.101 *N* in the mold group. The difference was statistically significant (*p* < 0.05)(Fig. 8). The force in the chicken tendons was 260.647 ± 22.161 *N* for the mold group and 243.552 ± 15.881 *N* for the pocket group. Failure loads for all freezing methods were significantly higher than that of the non-freezing treatments groups (Table 1, Fig. 8). In addition, the failure load of porcine tendon in the mold group was significantly higher than that in the pocket group (Fig. 8).

**Table 1:** Failure mode and failure load (mean ± standard deviation.) of porcine and chicken’s tendons with different treatments

Group		Porcine (*n* = 45) Failure load (*N*)	Failure mode	Chicken (*n* = 45) Failure load (*N*)	Failure mode
Control group		135.654 ± 14.140	Avulsion	113.780 ± 10.135	Avulsion
Unfrozen treatment	Gauze group	74.310 ± 12.707	Slippage	63.903 ± 14.616	Slippage
	Suture group	144.991 ± 21.064	Avulsion	101.774 ± 16.623	Avulsion
Frozen treatment	Pocket group	1746.337 ± 68.849	Slippage or fracture inside the clamp	260.647 ± 22.161	Fracture near the central point
	Mold group	2121.651 ± 73.101	Fracture near the central point	243.552 ± 15.881	Fracture near the central point

## Discussion

In the presented study, the effects of different fixation methods for clamping tendons of different diameters were compared. The results showed that the avulsion or slippage around the clamping area was found in control group (general metal clamp) and unfrozen groups (gauze and suture groups) for both chicken and porcine tendon, while rupture near the central point occurred in frozen groups with the exception of porcine tendons in the pocket group. These data suggest that the ordinary conventional clamp may achieve a higher gripping force with freezing methods; however, the pocket method was only effective for smaller diameter tendons. In order to meet the demand of a high clamping force for large diameter tendon, an application of the mold method may be beneficial.

Non-freezing clamps are often considered an inferior method to freezing clamps for tendon fixation, mainly due to the viscoelastic characteristics of the wet soft tendons making it hard for the clamps to grip the tendon tightly [4]. The extrusion during the loading process increases the moisture content of the holding part, and spraying of saline to maintain the moisture of the tendon surface to avoid change in the mechanical properties of the tendon [17] may further reduce the friction between the clamp and tendon. Besides, due to the deformation of the tendon at the clamp line, uneven stress distributions can lead to more damage in the clamping area [18]. The relative displacement between the outer layer of the tendon (which is well fixed with the clamp) and the inner layer of the tendon can also create avulsion. The gauze treatment was even worse than untreated control tendons, because of decreased friction between the gauze and the tendon. In the suture group, the failure load was similar to the control group. This suggests that the conventional clamp with non-freezing methods could not provide enough grip strength for mechanical tendon testing.

Freezing clamps can fixate the tendon firmly and decrease injuries to the clamping area of the tendon because the frozen tendon is tightly attached to the clamp and is difficult to deform. Freezing can increase the friction coefficient between the clamp and the tendon, and the hardness of the clamping area. As a result, the effectiveness of clamping is increased, and the damage caused by the clamp’s teeth to the tendon and the displacement between the outer and inner layers of the tendon was reduced [5]. The freezing clamp was first proposed by Riemersa and Schamhardt in 1982 and was called the ‘cryo-jaw’ [19]. They expected to prevent the deformation of tissues in high loads by freezing the tendons with clamps with expansion of liquid CO2. In 2004, Wieloch et al. [20] soldered liquid nitrogen containers consisting of copper plates to the clamps. Ramachandran et al. [21] also designed a special freezing assembly that included a container to circulate coolant, the nitrogen and ice container to fixate the specimen. However, this costly and time-consuming method is not widely available and is also difficult to design and manufacture, so more affordable, efficient, and easy to operate methods are worth exploring. Recently, Hangody used dry ice pockets to freeze a modified clamp called Shi’s clamp to obtain a relatively satisfactory outcome. We applied Hangody’s method but used conventional metal clamps with dry ice (Fig. 6). Although slippage occurred in the larger diameter porcine tendons, the failure load significantly increased compared with non-freezing treatments. The reason may be that the fixation ability of our clamp was lower than the specially designed Shi’s clamp and unable to provide enough grip strength to reach the rupture point of porcine tendons. However, the slippage or avulsion around the clamp line was not be observed in the pocket group of smaller chicken tendons which requires lower load to reach structure failure. This proves that Hangody’s pocket method works, but only to a limited extent. In the mold group, which is an improvement of Hangody’s pocket method, rupture was only observed where failure mode occurred near the central point in both porcine and chicken tendons. Moreover, porcine tendons in the mold group reached the failure threshold with significantly higher loads than tendons in the pocket group. However, the difference was not observed in chicken tendons. These findings suggest that our mold method works for both large- and small-sized tendons, while conventional clamps pocketed with dry ice are only effective for small-size tendons. Our method was especially effective in the test of large-size tendons.

The customized mold strictly controls the position and direction of the clamps during freezing, which avoids the difficulty of clamp installation caused by tendon torsion and bending. Studies have shown that there is fiber stretching and sliding during tendon loading [2], but the fiber sliding of frozen tendons may be limited and the tendon may present a similar brittle failure behavior [22] in the failure testing, which will lead to abnormal data. Therefore, it is necessary to avoid the effects of freezing in the test area. Since running water does not freeze easily, in this study, a continuous flow of water was placed above the center of the tendon to avoid the test area of tendon freezing. Since the water was spread along the surface of the tendon, the spread range is related to the height, width and diameter of the water flow, which was adjusted according to the actual situation. After the liquid nitrogen infusion, it is necessary to continue water perfusion for 1 min to offset the secondary freezing of tendon by low-temperature clamps and avoid the possibility of insufficient non-frozen area for mechanical testing of the tendon. On the other hand, the water perfusion could not be too long to decrease the freezing effect on clamping area of tendons. Our preliminary findings did show inconsistent melting times under different surrounding temperature conditions. Accordingly, the temperature of the experimental environment was maintained at 18–20℃ for standardization.

In this study, we found several advantages of our conventional clamp frozen with our customized mold. First, it could provide enough holding strength for tendon mechanical test as high as 2000 *N*, which represents nearly 16 times enhancement over the control group. Moreover, this technique prevents the tendon test area from freezing and the possibility of further tissue properties changes caused by the frozen state. The slippage and avulsion in the clamping area did not occur in our mold method, but they occurred in all other groups. Secondly, the mold method demonstrated high efficiency. In the Hangody’s study [11], the frozen mothed of dry ice pocket took about 8 min which is similar to what we found in our pocket group. Scholze [7] reported their preparation time for 3D printing technology clamp would be 10–15 min. The cryo-jaw would spend further more time for preparation [7, 9]. On average, preparation for test of tendon was 5–6 min for our mold method, which would be time-saving and convenient for measuring large sample. Thirdly, the cost of our mold method was relatively low. The special customized clamp for roughening clamp surface that was used in other researches costs between 35 and 100 US dollars [8, 9]. The cryo-jaw would be more complicated and also cost more [4]. In our study, the non-special designed clamp, which came with the testing machine in the original package, and the poly foam box for making molds cost next to nothing. The only cost for our mold method is liquid nitrogen (approximate 0.4–0.5 US dollar per sample).

This study has some limitations. First, the experimental conditions of this study are affected by the type of clamps and the experimental environment. The clamping force of different types of clamps is different, and the perfusion time may be slightly different under various experimental environments. Second, the determination of slippage was done by the naked eye, and there may still be very small slippage that was not observed. This may cause a certain degree of error. Third, there was a large amount of liquid nitrogen and water dripping in the experimental operation of the mold group. Therefore, a better method to collect nitrogen and water separately and maybe recycle them should be considered for future study. This may reduce the cost even further.

## Conclusions

The freezing clamp could be a better choice for soft tissue clamping scheme. The templated freezing of clamp through a customized mold and liquid nitrogen could improve the effectiveness of gripping force for tendon’s biomechanical tests. This method is simple, high efficiency and affordable. Applying this method to non-special designed clamps can achieve a relatively satisfactory effect.

## Supplementary Information

**Additional file 1.** Warm water continued to flow after the liquid nitrogen stopped.

## Data Availability

All data generated or analyzed during this study are included in this published article [and its supplementary information files]. The authors did not receive support from any organization for the submitted work. The authors have no relevant financial or non-financial interests to disclose.
